# Insect reproductive behaviors are important mediators of carrion nutrient release into soil

**DOI:** 10.1038/s41598-021-82988-6

**Published:** 2021-02-11

**Authors:** Brooke K. Woelber-Kastner, Serita D. Frey, Daniel R. Howard, Carrie L. Hall

**Affiliations:** 1 College of Life Science and Agriculture, University of New Hampshire, Spaulding Hall Rm G37; 38 Academic Way, Durham, NH 03824 USA; 2 2415 Eisenhower Ave, Alexandria, VA 22314 USA

**Keywords:** Zoology, Ecology, Behavioural ecology

## Abstract

Current declines in terrestrial insect biomass and abundance have raised global concern for the fate of insects and the ecosystem services they provide. However, the ecological and economic contributions of many insects have yet to be quantified. Carrion-specializing invertebrates are important mediators of carrion decomposition; however, the role of their reproductive activities in facilitating this nutrient pulse into ecosystems is poorly understood. Here, we investigate whether insects that sequester carrion belowground for reproduction alter soil biotic and abiotic properties in North American temperate forests. We conducted a field experiment that measured soil conditions in control, surface carrion alone, and beetle-utilized carrion treatments. Our data demonstrate that *Nicrophorus* beetle reproduction and development results in changes in soil characteristics which are consistent with those observed in surface carrion decomposition alone. Carrion addition treatments increase soil labile C, DON and DOC, while soil pH and microbial C:N ratios decrease. This study demonstrates that the decomposition of carrion drives soil changes but suggests that the behaviors of insect scavengers play an important role in the release of carrion nutrients directly into the soil by sequestering carrion resources in the ecosystem where they were deposited.

## Introduction

Historic declines in terrestrial insects have been documented globally^1–3^. Among these declines, both specialist and generalist insect populations have been effected, with Coleoptera (beetles) and Lepidoptera (butterflies and moths) experiencing elevated annual rates of decline relative to other insect taxa^2^.These patterns of decline are well understood in temperate regions relative to tropical ecosystems^2,3^; however, our knowledge of susceptible insect groups is constrained to measurements of overall insect abundances and to species in well studied taxa and ecosystems. Regardless, these observed declines have raised concern among scientists regarding the potential impact that reduced insect populations may have on ecosystems. For instance, in addition to serving as a primary food source for a variety of organisms, insects provide other ecosystem services which are valued at approximately 57 billion USD annually^4^. However, insect effects are often overlooked based on their relative contribution to total biomass across ecosystems, particularly in comparison to plant and microbial biomass^5^. Yet, research has demonstrated that insects can have strong indirect effects on soil and nutrient availability^6–15^. Still, there remains a large gap in the literature with respect to how less well-studied insects and their behaviors modulate soil habitat and nutrient availability.

Across ecosystems, carrion serves as a long-lasting and concentrated source of nutrients^16^. Although the contribution of large carrion to soil nutrients and microbial biomass is well-documented^17–20^, few studies investigate small vertebrate carcasses and the role of individual insect behaviors in the release of these concentrated nutrients. Rather, the majority of studies document necrophilous insect succession patterns to understand how community assemblages contribute to carcass degradation^21–24^, with results indicating insect activity is essential to increasing decomposition rates^22,25–29^. Although it is recognized that insect behaviors are important contributors to decomposition, few studies have directly quantified how specific behaviors, such as those related to mating and reproduction, may contribute to soil nutrient cycling and the microbial community.

Burying beetles (Coleoptera: Silphidae: *Nicrophorus*) are well-suited for investigating how insects modulate carcass decomposition, as they scavenge and sequester carrion belowground and utilize it as a reproductive resource^30,31^. Species such as *Nicrophorus orbicollis* locate carcasses during flight by detecting volatized chemical cues, and immediately following carrion discovery, a male and female burying beetle pair will collaboratively work to bury the carcass to variable depths within the soil^30,32^. During burial, beetle pairs will strip the fur or feathers from the carrion and roll it into a mass of meat referred to as the brood ball^30–32^. Beetle pairs will copulate frequently during this time^33–35^, while also coating the carcass with oral and anal exudates containing antimicrobial compounds that delay microbial-mediated decomposition^10,36–39^. During carcass burial and preparation, females lay eggs in the surrounding soil^30–32^ and after approximately three to five days eggs hatch and larvae arrive on the carcass where *N. orbicollis* pairs provide biparental care to developing young^31,40^. Approximately 8–11 days following larval arrival on the carcass, parental care is terminated and larvae disperse in the surrounding soil, where they pupate, then eclose as adults^31,40^.

Recent research demonstrated that in forest habitats, burying beetles are able to sequester up to 75% of small vertebrate carrion (e.g., field mice) for reproduction^41^, indicating that beetles are one of the primary insect groups engaged with facilitating the decomposition of small carrion in forest ecosystems. However, although carrion sequestration by Nicrophorine burying beetles has historically and anecdotally been considered an essential process to facilitate nutrient release into soil^30,41^, recent research is the first to quantitatively support this, albeit in an artificial lab setting^42^. Here we describe an experimental field study designed to assess whether burying beetle reproduction affects soil nutrient cycling and microbial biomass relative to surface carrion decomposition alone (i.e., no insect involvement) in a northern deciduous forest. Our objective was to determine how beetle-mediated carcass burial and utilization affects soil abiotic and biotic properties in comparison to soils with no biological input and determine whether these changes were consistent to those observed during carrion decomposition in the absence of these insect behaviors.

## Results

### Soil abiotic characteristics

**Figure 1:**
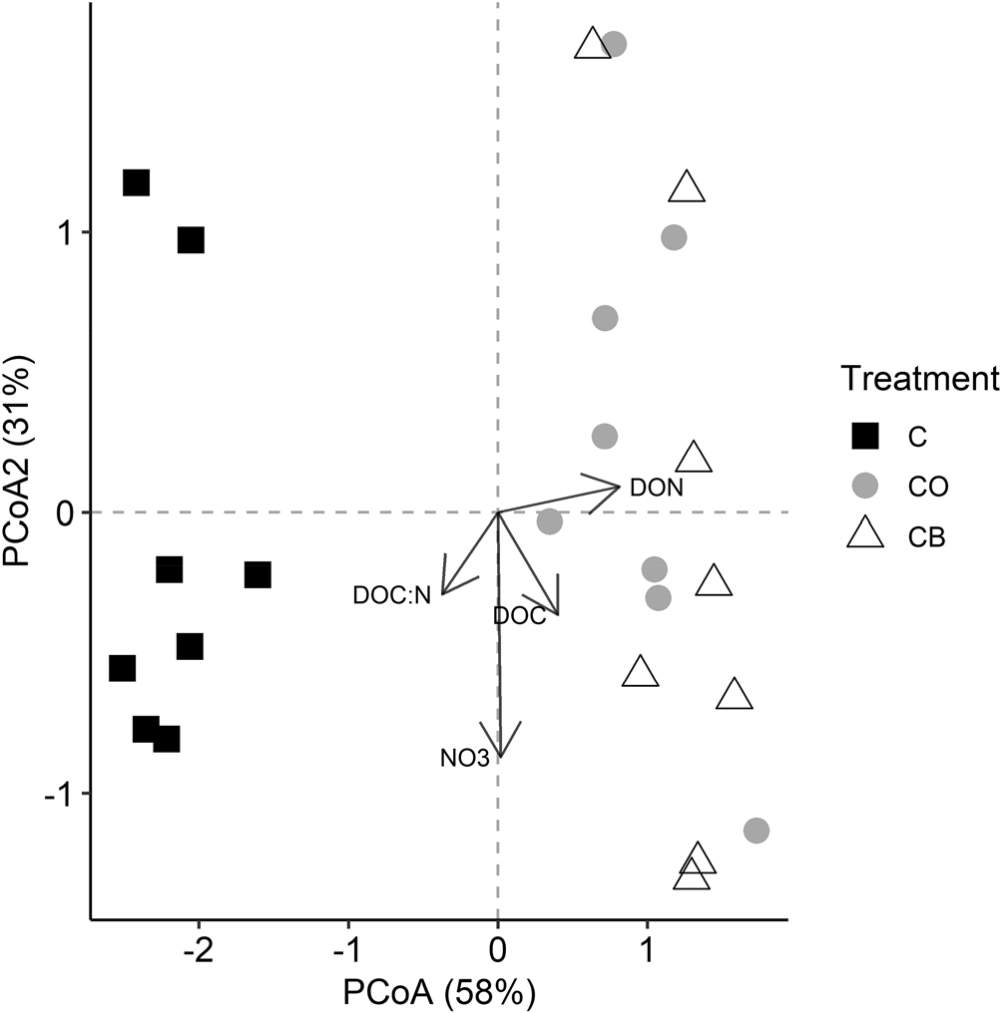
Principal coordinates axes of soil abiotic characteristics (pH, moisture, labile C, DOC, DON, DOC:N Ratio, inorganic N, total C:N Ratio) using Euclidean dissimilarities between samples. There was strong separation in soil abiotic characteristics in carrion addition plots relative to the control. Squares indicate controls (C), circles indicate carcass only (CO), and triangles indicate burying beetle plots (CB). Soil abiotic characteristics with a covariance greater than 0.3 were retained (indicated by arrows).

Principal coordinates analysis indicated that there was strong separation in soil abiotic characteristics between carcass addition study plots and control treatments along axis 1 (Fig. 1). The PCoA explained 89% of the total variation in soil abiotic characteristics, with 58% of the variation explained by axis 1. Axis 1 was largely explained by the covariances of soil DON, DOC and DOC:N, while soil NO_3_^−^ covaried with axis 2. PERMANOVA indicated that the observed separation between carrion addition treatments and the control within the ordination was significant (F_2,23_ = 5.02; R^2^ = 0.32; *P* = 0.001), as both the carcass only (CO) and carcass plus burying beetle (CB) treatments exhibited significantly different soil abiotic characteristics as compared to the control plots (*P* < 0.01). There was no difference in soil abiotic characteristics between the carcass only and carcass burying beetle treatments.

Subsequent analysis of variance indicated an effect of treatment on soil pH (F_2,21_ = 91.03; *P* < 0.001), with Tukey’s mean separation indicating that the burying beetle plots were significantly more basic than both the carcass only (*P* < 0.01) and control treatments (*P* < 0.001), while carcass only treatments were significantly more basic than the control soil (*P* < 0.001; Table 1). However, the observed difference between the carcass only and carcass burying beetle treatments was relatively small relative to the difference between the control and carrion addition treatments (Table 1). Treatment did not influence soil moisture (F_2,21_ = 1.44; *P* = 0.26), and there was no effect of treatment on soil inorganic N levels.

**Table 1:** Soil characteristics (means ± 1SE; n: C = 8, CO = 8, CB = 8).

Treatment	Control (C)	Carcass only (CO)	Carcass burying beetle (CB)	*P*-value
pH	4.9 ± 0.07	6.2 ± 0.09***	6.7 ± 0.13***^●^	** < 0.001**
Moisture	0.56 ± 0.06	0.66 ± 0.08	0.68 ± 0.04	0.260
Labile C (µg C g^−1^ soil)	2456 ± 308	3520 ± 735	4402 ± 479*	**0.018**
DOC (µg g^−1^ soil)	512 ± 63	1570 ± 704**	2513 ± 424***	** < 0.001**
DON (µg g^−1^ soil)	73 ± 8	1473 ± 114***	1582 ± 103***	** < 0.001**
DOC:DON	8.1 ± 0.34	1.1 ± 0.39**	1.8 ± 0.27*	** < 0.001**
NH_4_^+^ (µg N g^−1^ soil)	43.9 ± 3.9	40.8 ± 3.9	40.7 ± 4.9	0.820
NO_3_^−^ (µg N g^−1^ soil)	4.05 ± 1.08	3.79 ± 0.89	4.51 ± 1.41	0.904
Total C (%)	11.8 ± 0.88	11.6 ± 1.04	11.6 ± 0.86	0.980
Total N (%)	0.58 ± 0.06	0.69 ± 0.07	0.73 ± 0.05	0.180
C:N	21.2 ± 1.28	17.02 ± 0.80*	16.1 ± 0.85**	**0.004**
MBC (µg g^−1^ soil)	2409 ± 102	2469 ± 346	2972 ± 396	0.385
MBN (µg g^−1^ soil)	306 ± 35	1301 ± 279***	1632 ± 190***	** < 0.001**
MBC:N	10.1 ± 1.18	2.5 ± 0.38***	2.1 ± 0.18***	** < 0.001**

Effects of treatment were tested with a one-way ANOVA. Treatment level differences were determined by Tukey-pairwise comparisons.

* significant at *P* < 0.05; **P < 0.01; ***P < 0.001 relative to the control.

^●^ significant at *P* < 0.05 relative to carcass only treatment.

Bolded *P*-values indicate a significant ANOVA result.

**Figure 2:**
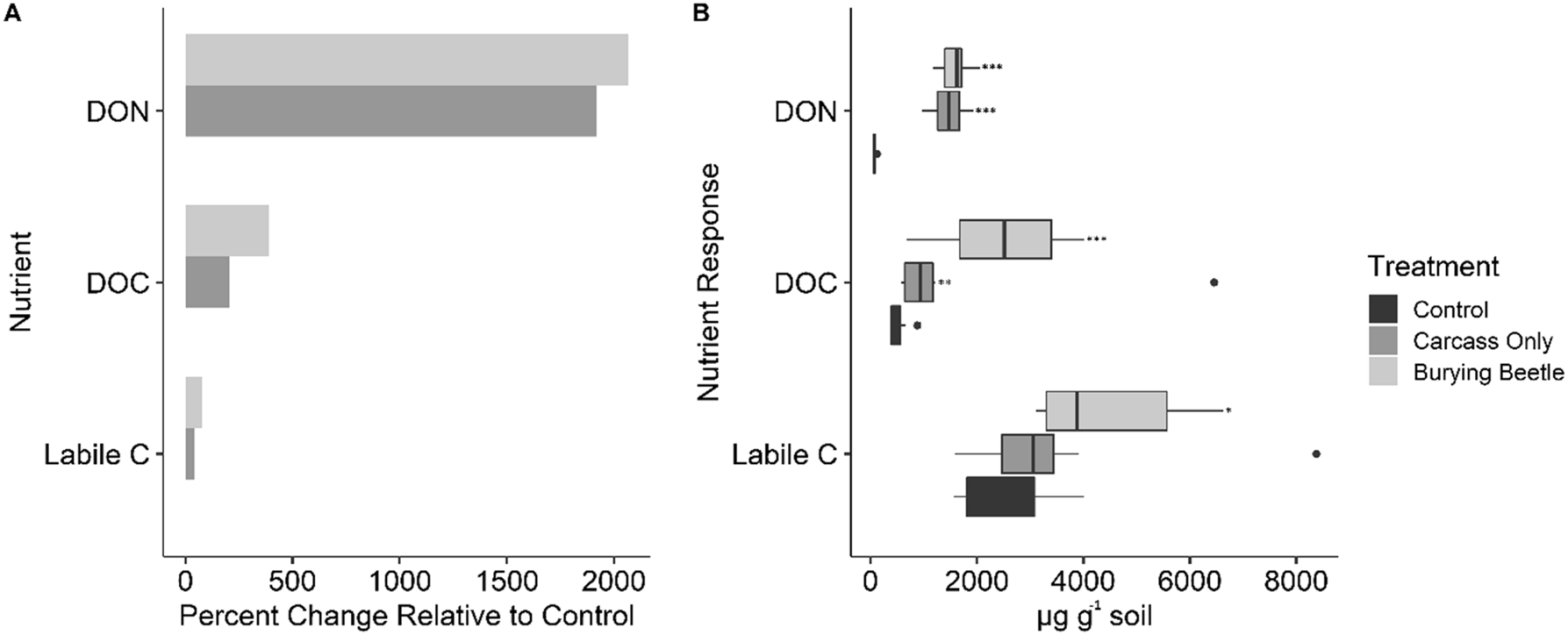
(**A**) Percent increase in each soil nutrient relative to the control and (**B**) changes in soil nutrient levels according to treatment. Dark grey indicates the control, gray indicates the carcass only treatment, and light grey indicates treatments with both carrion and burying beetles. Nutrient abbreviations: Dissolved organic nitrogen (DON), Dissolved organic carbon (DOC) and Labile Carbon (Labile C). Boxplot elements are as follows: center line, meidan; box limits, upper and lower quartiles; whiskers, 1.5 × interquartile range; points, outliers. Levels of significance relative to the control is indicated as follows: * significant at *P* < 0.05; ** *P* < 0.01 and *** *P* < 0.001.

Soil C mineralization, an index of bioavailable C, differed among treatments (F_2,21_ = 4.93; *P* < 0.05) (Table 1, Fig. 2). Specifically, the burying beetle treatments exhibited a significantly greater labile C pool compared to the controls (*P* < 0.05). Soil dissolved organic carbon (DOC; non-fumigated samples) also significantly differed among treatments (F_2,23_ = 17.59; *P* < 0.001), with both the CO (*P* < 0.01) and CB (*P* < 0.001) treatments exhibiting greater DOC than controls (Table 1, Fig. 2). With respect to dissolved organic nitrogen (DON), carcass addition plots exhibited significantly greater levels of DON than the controls (F_2,21_ = 21.87; *P* < 0.001; Tukey HSD test: *P* < 0.001) (Table 1, Fig. 2). These changes in DOC and N resulted in significant decreases in the ratio between DOC:N (F_2,21_ = 114.38; *P* < 0.001) in the CO (*P* < 0.01) and CB treatments (2018: *P* < 0.05) compared to controls. The effect of treatment on total soil C:N ratio was consistent with these findings, with the controls exhibiting significantly greater soil C:N ratios relative to the CO (*P* < 0.05) and CB treatments (*P* < 0.01). However, there was no difference in total C (F_2,21_ = 0.019; *P* = 0.98) or N (F_2,21_ = 1.86; *P* = 0.18) among treatments.

### Microbial biomass and community composition

Analysis of variance indicated microbial biomass N was greater (F_2,21_ = 28.48; *P* < 0.001) within both the CO and CB treatments relative to the controls (*P* < 0.001). Microbial biomass C did not differ among treatments (F_2,21_ = 0.997; *P* = 0.386) resulting in an MBC:N ratio that was significantly reduced in both the CO and CB treatments relative to the controls (*P* < 0.001).

Microbial community composition was not different across treatments (F_2,21_ = 1.28; *P* = 0.297; Supplementary Fig. S1 & Table S1) nor was there a difference in the relative biomass of individual microbial groups or the fungal:bacterial biomass ratio (Table 2).

**Table 2:** Soil microbial biomass as estimated by phospholipid fatty acid (PLFA) analysis (nmol g^*−*1^ dry soil) (means ± 1SE; n: C = 8, CO = 8, CB = 8).

Treatment	Control (C)	Carcass Only (CO)	Carcass Burying Beetle (CB)	P-value
Total microbial biomass	185 ± 26	235 ± 38	258 ± 33	0.298
Fungi	29.92 ± 3.59	38.7 ± 4.79	40.9 ± 5.08	0.214
Bacteria	141 ± 21	199 ± 31	180 ± 26	0.306
Fungi:bacteria Ratio	0.225 ± 0.02	0.226 ± 0.02	0.219 ± 0.01	0.96

Effects of treatment were tested with a one-way ANOVA.

* significant at *P* < 0.05 relative to the control.

## Discussion

The rate of carrion decay and break-down is facilitated by necrophilous insect groups^21,23^, and these activities provide a localized, but concentrated, nutrient pulse into the soil^16,43,44^. Our hypothesis that burying beetles would affect soil characteristics in response to their reproductive activities was not supported. Rather, the results of our study demonstrate that carrion attributes drive soil characteristics in response to decomposition. However, the reproductive activities of burying beetles and consumption of the carcass by developing larvae did not negate the benefits of carrion decomposition, as the changes in soil characteristics within the burying beetle treatment were consistent with those observed during surface carrion decomposition alone. Taken together, these findings indicate that the carrion decomposition process drives alterations in soil characteristics, and that burying beetle reproduction does not appreciably reduce carrion contributions to soils. For instance, we found that there was an increase in soil pH (pH > 6) and dissolved organic C and N in carrion addition plots, regardless of burying beetle utilization of the carcass for reproduction and larval growth. Changes in soil pH during carcass decomposition are well-documented^18,20,45^, with increases associated with the influx of by-products released during tissue deterioration, most commonly driven by increases in soil ammonium^25,46,47^. Although we did not observe increased soil NH_4_^+^ in the carcass only (CO) nor the burying beetle (CB) treatments, the observed increases in dissolved organic C and N align with nutrient profile changes observed in Keenan et al. (2018). These findings indicate that although the carrion was consumed by burying beetle larvae in the burying beetle treatment, the observed abiotic changes were consistent with microbially mediated carcass decomposition on the soil surface and still result in a net contribution of nutrients to the soil. However, the release of nutrients between these two treatments is likely facilitated through differing metabolic pathways, with insect frass within the burying beetle treatment potentially serving as the primary source of N relative to a naturally decomposing carcass^14,48,49^.

Insect necrophilous behaviors co-occur with shifts in microbial community abundances in response to the stage of decomposition^47,50^. However, we did not observe any differences in microbial abundance and biomass in response to carrion only nor burying beetle treatments. Rather, there was a significant reduction in microbial biomass C:N ratio from 8:1 within the control to approximately 2:1 in the carrion addition treatments, which is lower than ratios typically reported in soils without carrion inputs^51^. Microbial biomass N increased within these treatments, while microbial biomass C did not differ. The increase in microbial biomass N is likely explained by the influx in soil N within both the carcass only and burying beetle treatments. Following nutrient release within these treatments, microbes likely immobilize N and differentially store nutrients which would alter their biomass C:N ratios^52–55^. Additionally, the greater abundance of bacteria across our plots relative to fungi may also contribute to these low ratios, as bacteria tend to exhibit lower C:N ratios (4:1 to 10:1) than those observed in fungi (8:1 to 29:1)^56^. This data indicates that the incorporation of carrion in ecosystem landscapes, and the sequestration and consumption of these carrion by burying beetles for reproduction and larval growth, create nutrient hotspots in the soil which are utilized during microbial metabolism. However, further studies are required to provide increased resolution regarding the associated stoichiometric ratios of carrion associated microbial communities, and the role of necrophilous groups in isolating carrion and creating nutrient hotspots in the soil.

The indirect benefits of insect driven decomposition of carrion are often underappreciated and understudied relative to other detrital inputs. As carrion size decreases, invertebrates are increasingly likely to utilize the resource for their own life histories^57,58^. Indeed, research suggests that the activities of insect scavengers play an essential role in preventing vertebrate scavengers from removing carrion from the ecosystem, as the proportion of carrion removed by vertebrate scavengers is greatly reduced in warm weather (from 65 to 16–20%) when invertebrates are active^57,59,60^. Burying beetles alone can sequester greater than 65% of small carrion in forest ecosystems, compared to 10–35% by vertebrate scavengers^41,61^. In this context, our study indicates that at the ecosystem scale, insects which sequester and isolate resources from other scavengers can play a significant role in creating nutrient hotspots where an organism died. When we consider insect groups such as the burying beetle (75 species in Northern Hemisphere), that can sequester vertebrate carcasses ranging in size from 4 to 210 g^31^, and are distributed across North America with temporal and phenological shifts in activity patterns^31,62,63^, the potential nutrient input from carrion sequestration can be significant. For example, in NH forests alone there are five burying beetle species active May–September. If we presume 6% death rate per week^64^ of small mammals (~ 35 g), captured across two weeks in the summer^65^, 75% of which are used by burying beetles^41^, while assuming this mortality and capture rate is consistent across 16 weeks of activity, their behaviors could contribute up to 2.37 g DON or 2.14 g DOC m^−2 ^y^−1^. This is a conservative estimate given these calculations do not account for utilization of carcasses of greater mass.

Given the limitations of field-based studies that examine ecosystem interactions and the limited nature of our understanding of invertebrate decomposer roles in modulating soil properties, future studies would do well to further investigate the relationships among invertebrate scavengers and soil nutrients. As our study prevented invertebrate activities on surface carrion treatments, we were unable to draw conclusions regarding the significance of burying beetle reproduction and larval development on soil nutrients relative to other invertebrate scavenger activities. Future studies could further investigate the role that carrion sequestration by burying beetles, larval activities, and larval number and/or mass, play in creation of nutrient hotspots and how it influences the lateral and vertical spread of nutrients relative to surface-level carrion decomposition. Additionally, it would be informative to measure the proportion of carrion nutrients that are retained within larval biomass versus released into the soil via metabolic pathways such as insect frass, elucidating the chemical and nutrient characteristics of larval frass within scavenging insect groups.

## Methods

### Burying beetle collection and maintenance

*Nicrophorus orbicollis* was captured in the summer of 2018 at the University of New Hampshire’s (UNH; Durham, NH, USA) forest sites located at Kingman and Woodman Farms using 5-gallon above-ground pitfall traps baited with aged chicken liver^66,67^. Wild-caught beetles were maintained in the lab for approximately 2 weeks in solitary acrylic containers (Pioneer Plastics, 109.53 mm × 57.15 mm × 44.45 mm) filled with moist peat. Beetles were maintained under a 14:10 day:night light cycle, provided water ad libitum, and fed raw pork loin twice weekly.

### Experimental design

To determine the effect of carcasses and burying beetle reproductive behaviors on soil biotic and abiotic properties, field site treatments were set up during the summer of 2018. Plots were established in a mixed deciduous forest at UNH Kingman Farm at the onset of the *N. orbicollis* reproductive season in June. Dominant trees species at the site are Red Maple (*Acer),* Black Oak (*Quercus*)*,* American Beech (*Fagus*)*,* Ash (*Fraxinus*), and Birch (*Betula*). Eight (1 m^−2^) plots were established approximately 30 m apart. Each plot contained the following treatments: no input (i.e., control, C), carcass only (CO), and a carcass with a burying beetle pair (CB). Based on lower than expected burying beetle breeding success rates in the field from a preliminary study (see supplementary materials for preliminary study design and results)^61,68,69^, there were six replicates per plot for the burying beetle (CB) treatment, while there were three replicates per plot for each of the control (C) and carcass only (CO) treatments, with treatment replicates within a plot later homogenized. To confine the burying beetles to one carcass and prevent other insects from accessing it, each carcass (previously frozen, freshly thawed mouse; 31.57 g ± 1.41; Rodent Pro, Evansville, Indiana, USA) was placed within an aluminum mesh enclosure (10 cm^−3^) containing soil excavated from the plot from which visible invertebrates and fine roots were removed. Enclosures were installed within the organic horizon to the depth of the mineral soil (~ 5 cm). They were placed in rows, with each individual enclosure separated by 10 cm. Following placement of enclosures, the entire plot was covered with a barrier constructed of galvanized metal hardware cloth (½ in × ½ in) that was secured in place with wooden stakes to prevent vertebrate scavengers from disturbing the plot.

Each enclosure was destructively sampled 21 days following its placement in the field. Samples from each enclosure (i.e., soil, carcass, larvae) were placed in an individual zip-top plastic bag and chilled in a cooler during transport back to the laboratory. On the same day of collection, samples were sieved (< 2 mm) to remove coarse roots (> 2 mm), rocks, carcass remains, and burying beetle larvae. Fine roots (< 2 mm) remained in the sieved soil sample. For CO and CB treatments, the final mass of the carcass was recorded. In addition, reproductive success (offspring production) or failure (no offspring) was recorded for CB treatments. Following soil processing, all replicates from the same treatment within a plot were combined and homogenized. The only exception to this occurred when CB treatments failed to produce offspring (current study *N. orbicollis* successful breeding rates: ~ 66%). In these situations, CB treatment replicates which produced offspring were homogenized, while any failed CB treatments within that same plot were omitted, as the focus of this study was on successful burying beetle reproduction. Following homogenization, all soil samples were stored at 4 °C until further lab analyses were performed for approximately 1 week. Following treatment homogenization, final treatment sample sizes (n) were as follows: C = 8, CO = 8, CB = 8.

### Soil chemical analyses

Soil moisture was determined gravimetrically by drying subsamples (~ 5 g) at 60 °C for 48 h. Soil pH was evaluated using a digital pH probe (Cole-Palmer, Vernon Hills, IL) in a 1:10 soil: water suspension. Inorganic N was determined by extracting soil with 2 M KCl (1:5 wt:vol), filtering (#40 Watman filters), and quantifying the concentration of nitrate (NO_3_^−^ ) and ammonium (NH_4_^+^) calorimetrically using a multi-detection microplate reader (Synergy™ HT, BioTek Instruments, Inc., Part #7091000, Winooski, Vermont, USA) at 540 and 640 nm, respectively. Nitrate quantification was determined by the vanadium (III) reduction reaction^70^ while ammonium was determined using the indophenol-blue method^71^. The detection limits for both NH_4_^+^ and NO_3_^-^ was 0.1 ppm^72^.

Total soil C and N were determined by dry combustion of finely ground samples using a Costech C/H/N/S Elemental Analyzer. Labile C (carbon mineralization) was estimated using a 30-day incubation with soil subsamples (10 g) sealed in 0.933 L Mason jars incubated at 25 °C. Headspace samples were collected daily to determine atmospheric CO_2_ concentrations using a LI-COR infrared gas analyzer (Model LI-6252, LI-COR Biosciences, Lincoln, NE). Jars were flushed with CO_2_-free air following each headspace sampling to maintain O_2_ levels.

### Microbial analyses

Microbial biomass C and N were determined on 0.5 M K_2_SO_4_ soil extracts (1:3 wt:vol) following chloroform fumigation^73–75^. Dissolved C and N concentrations in the extracts were determined using thermal oxidation with near infrared carbon detection followed by chemiluminescence nitrogen detection on a Shimadzu TOC-L with an attached TNM-L unit.

Microbial community composition was determined using phospholipid fatty acid (PLFA) analysis. Microbial lipids were extracted from 1 g of sieved, root-free freeze-dried soil that had been stored at − 80 °C until analyses began. Lipids were extracted by utilizing a single-phase solvent (chloroform) combined with phosphate buffer which was based on a modified Bligh and Dyer (1959) extraction procedure^76–78^. This technique extracts lipids from viable microorganisms captured at the time of sampling. Lipid extracts were fractionated on silicic acid columns into neutral, glycol- and polar lipids, with only polar lipids collected. Following collection, polar lipids were methylated with 0.2 M methanolic KOH solution to form fatty acid methyl esters (FAMEs). FAMEs were dried and reconstituted in hexane for quantification on a Varian 3800 GC-FID (Varian, Inc., Walnut Creek, CA). FAME peaks were compared against a standard library of FAMEs and based on retention time data of the known standards. Peak area concentrations were converted to nmol PLFA g^−1^ dry soil based on the peak area of its matching standard peak. The polyenoic unsaturated fatty acids, 18:2w6 and 18:1w9c, were considered fungal biomarkers^79,80^. Branched, saturated gram-positive fatty acids of i15:0, a15:0, i16:0, i17:0 and a17:0 as well as the monoenoic and cyclopropane unsaturated gram-negative fatty acids of 16:1w7c, 16:1w7t, 18:1w7c and cy19:0 were considered part of the total bacterial biomass^81,82^. Total bacterial biomass was also represented by 15:0, which was considered a general bacterial marker to complete the bacterial assessment^82^.

### Statistical analyses

All data analyses were performed in R version 3.5.3 (R Core Team, 2019). To assess the multivariate response of soil abiotic characteristics (pH, moisture, inorganic N, total C:N ratio, DON, DOC and DOC:N ratio) to treatment, a principal coordinate analysis (PCoA) was conducted with treatment means in the package *ape*^83^. To determine whether the visualized separation of treatments was significant, a Permutational Analysis of Variance (PERMANOVA) with Euclidean distance was conducted using the package *vegan*^84^. The function *betadisper* was used to determine whether data met the assumption of treatment homogeneity. Following a significant result within the PERMANOVA, pairwise comparisons amongst treatment groups were conducted with the package *RVAideMemoire*^85^.

Following a significant output from the PERMANOVA, univariate analyses were conducted to understand the response of individual abiotic response variables to treatment. Data were analyzed with a one-way ANOVA with treatment as the fixed effect with the package *car*^86^. Response variables included soil abiotic characteristics (pH, moisture, labile carbon, DOC, DON, DOCN Ratio, inorganic N, total C:N Ratio), and soil microbial biomass (MBC, MBN, MBCN Ratio, PLFA total microbial biomass, PLFA fungi, PLFA total bacterial biomass, PLFA fungi: bacteria ratio and PLFA gram-negative bacteria, gram-positive bacteria and general bacteria). Data were checked for normality and homogeneity of variance prior to analyses and transformed to better meet the assumptions of the analyses when necessary. To better fit the model assumptions, DON, DOC, and microbial biomass N were rank transformed while soil labile C was log transformed. Microbial biomass C:N ratio was square root transformed prior to analyses. Following a significant ANOVA, a Tukey HSD test was utilized to compare group means with the package *agricolae*.

Microbial community composition (based on the relative abundance of PLFA markers) was assessed using nonmetric multidimensional scaling analysis (NMDS) with Bray–Curtis distance in the package *vegan*. To determine abiotic soil characteristic relationships with the PLFA community, the function *envfit* was used and abiotic characteristics with a *P*-value of 0.1 or less were retained on the ordination. Following construction of the NMDS, a PERMANOVA based on Bray–Curtis distance was conducted by using 999 permutations to assess differences in microbial PLFA abundances in response to treatment based on their standard error with the function *adonis*. We also used the function *betadisper* to check for homogeneity of multivariate dispersion across factors. If the results of the PERMANOVA were significant, pairwise comparisons amongst treatment groups were conducted with the package *RVAideMemoire*. In order to understand whether the matrix of multivariate abiotic characteristics was significantly correlated and driving the changes in the microbial community composition matrix within each treatment, a Mantel test was performed with the package *vegan*. All treatments had a sample size of eight each.

## Supplementary Information

Supplementary Information.

## Data Availability

The data that support the findings of this study are available from the corresponding author upon reasonable request.

## Code availability

R code utilized in this study is available upon reasonable request from the corresponding author.
