# Supplementary material for: Insect reproductive behaviors are important mediators of carrion nutrient release into soil
Source: Sci Rep. 2021 Feb 11;11:3616. doi: 10.1038/s41598-021-82988-6 (PMC7878738; doi:10.1038/s41598-021-82988-6)
Supplement: Supplementary file 1 — Supplementary Information. [file 41598_2021_82988_MOESM1_ESM.docx]

**Title:** Insect reproductive behaviors are important mediators of carrion nutrient release into soil

**Author List:** Brooke K. Woelber-Kastner^1^, Serita Frey^1^, Daniel R. Howard^1^, and Carrie L. Hall^1^

**Author Affiliations:**

^1^College of Life Science and Agriculture, University of New Hampshire, Durham, New Hampshire USA

**Corresponding Author:** Brooke K. Woelber-Kastner, Spaulding Hall Rm G37; 38 Academic Way, Durham, NH 03824, [bw1054@wildcats.unh.edu](mailto:bw1054@wildcats.unh.edu)

SUPPLEMENTARY MATERIAL

**Supplementary Figure S1:** NMDS ordination of microbial community abundances by treatment as measured by phospholipid fatty acid (PLFA) analysis. There was no difference in the microbial community among treatments, and soil pH significantly correlated with microbial community abundances. Squares indicate control (C), circles indicate carcass only (CO), and triangles indicate burying beetle plots (CB). Soil abiotic characteristics with a correlation of 0.1 or greater were retained (indicated by arrows).

**
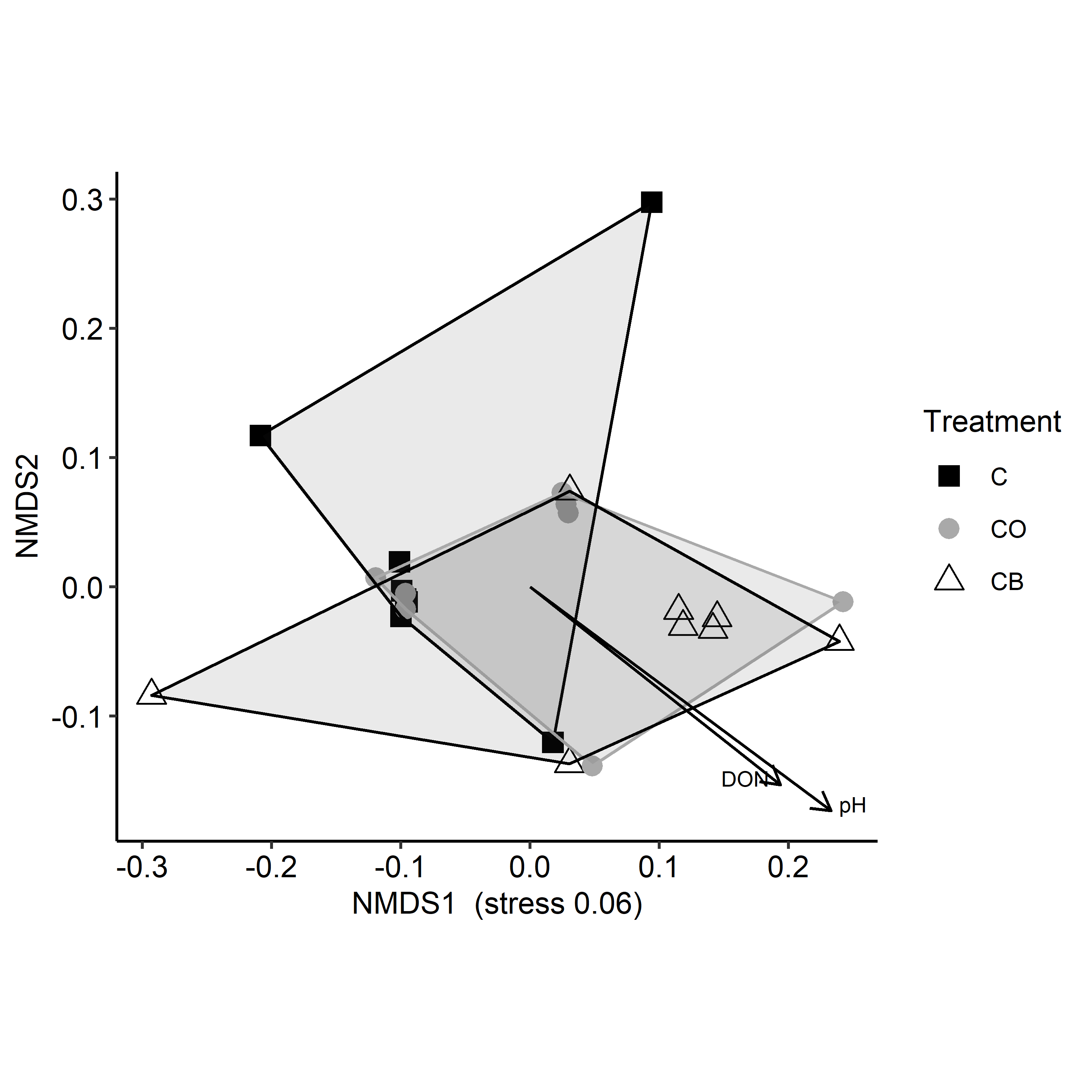
**

**Supplementary Table S1:** Soil abiotic attribute correlations with NMDS ordination axes.

|  | **2018 Environmental Correlations** | | | |  |
| --- | --- | --- | --- | --- | --- |
| **Response Variable** | *NMDS1* | *NMDS2* | *r2* | *pr(>r)* | |
| Labile C | 0.388 | 0.921 | 0.018 | 0.815 | |
| NO_3_^-^ | -0.828 | 0.559 | 0.027 | 0.742 | |
| NH_4_^+^ | -0.652 | -0.757 | 0.0003 | 0.996 | |
| Moisture | -0.827 | 0.561 | 0.017 | 0.826 | |
| pH | 0.619 | 0.785 | 0.332 | **0.020 *** | |
| Soil C:N | 0.039 | -0.999 | 0.078 | 0.443 | |
| DOC | 0.237 | 0.971 | 0.116 | 0.283 | |
| DON | 0.609 | 0.793 | 0.233 | 0.071 | |
| DOC:DON | -0.356 | -0.934 | 0.191 | 0.104 | |

SUPPLEMENTARY METHODS: PRELIMINARY STUDY

*Experimental Design*

In the summer of 2017, five forest sites owned by the University of New Hampshire (UNH) were used, with three plots within each site to account for soil heterogeneity. Hardwood forest ecosystems in this study included: 1) College Woods (*43°07’ 54.12” N, 70° 56’ 55.12” W*), 2) East Foss Farm (*43° 07’ 20.71” N, 70° 56’ 09.09” W*), 3) Kingman Farm 1, 4) Kingman Farm 2, (*43° 10’ 08.61” N, 70° 55’ 49.53” W*), and 5) Woodman Farm (*43° 09’ 04.61” N, 70° 56’ 31.02” W*). Plots were established at the beginning of July, which is near the end of *N. oribicollis* reproductive season. During the preliminary study, approximately 24% of burying beetle breedings were successful (e.g. produced offspring), which is lower than previously documented breeding success in field studies, although success rates are highly variable (Trumbo, 1990; Trumbo, 1995; Wilson & Fudge, 1984). Given the high burying beetle reproductive failure rate, we utilized only one field site the following summer (UNH Kingman Farm), and initiated field experiments at the onset of their peak active season (beginning of June). For the summer of 2017, each subplot contained three replicates of each treatment: no input (i.e. control, C), carcass only (CO), and a carcass with a burying beetle pair (CB).

*Statistical Analyses*

In the summer of 2017, total treatment samples sizes were as follows: C = 15, CO = 15, and CB = 7. Analyses conducted on preliminary multivariate responses of soil abiotic characteristics and microbial community followed those mentioned for the summer of 2018. However, for preliminary data each treatment was nested within field site.

Following a significant soil abiotic PERMANOVA, preliminary data were analyzed using a nested analysis of variance (ANOVA). The effect of field site on response variables was nested within the effect of treatment. Given this, field site is the level of replication and can be considered a random effect nested within the fixed effect of treatment. Data were checked for normality and homogeneity of variance prior to analyses and transformed to better meet the assumptions of the analyses when necessary. Soil NH_4_^+^, DOC:N , DON, DOC, and MBN were rank transformed, while labile carbon, MBC:N and total gram-positive bacteria were log-transformed.

SUPPLEMENTARY RESULTS: PRELIMINARY STUDY

*Soil Abiotic Characteristics*

Preliminary principal coordinates analyses indicated that there was strong separation in soil abiotic characteristics between carcass addition treatments relative to control treatments along axis 1. For the preliminary study conducted in 2017, axis 1 explained 58% of the variation and axis 2 explained an additional 31% of the variation in the data (Supplementary Fig. 2). The soil characteristics of DOC:N, DON, and DOC largely explained the variation in axis 1, while NO_3_^-^, DOC, and DOC:N covaried with axis 2. Results of the PERMANOVA indicated that both the carcass only (CO) and burying beetle (CB) treatments exhibited significantly different soil abiotic characteristics as compared to the control plots in the preliminary data (F_2, 37_ = 68.8; R^2^ = 0.57; *p* < 0.001; Tukey HSD test: p < 0.01).

Analyses of variance indicated a significant effect of treatment on soil pH (2017: F_2,23_ = 22.7; *p* < 0.001), with carcass addition plots (CO & CB) having significantly higher pH than control plots (*p* < 0.001) (Supplementary Table 2). Soil moisture did not differ across treatments in the preliminary study (F_2,23_ = 1.54; *p* = 0.25). With respect to soil nutrients, there was a significant effect of treatment on soil NH_4_^+^ levels (F_2,23_ = 4.38; *p* < 0.05) with significantly reduced levels of NH_4_^+^ in the CB treatments relative to the control (*p* < 0.05). Soil NO_3_^-^ levels did not differ among treatments (F_2,23_ = 0.252; *p* = 0.78).

During the preliminary study, there was no effect of treatment on soil carbon mineralization (measured as soil respiration) (F_2,23_ = 2.98; *P* = 0.088), but soil dissolved organic carbon (non-fumigated samples) significantly differed among treatments (F_2,23_ = 7.06; *P* < 0.01). Specifically, soil dissolved organic carbon was elevated in both the CO (*P* < 0.001) and CB (*P* < 0.001) treatments compared to the control (Supplementary Table 2). This same effect was observed with respect to dissolved organic nitrogen, (F_2,23_ = 27.09; *P* < 0.001) as carcass addition plots exhibited significantly higher levels relative to the control (*P* < 0.001). These changes in dissolved organic carbon and nitrogen resulted in significant decreases in the ratio between dissolved organic carbon: nitrogen (F_2,23_ = 31.86; *p* < 0.001) in the CO (*P* < 0.001; 2018: *P* < 0.01) and CB treatments (*P* < 0.001) compared to the control. The effect of treatment on soil total carbon: nitrogen ratio was consistent with these findings (F_2,23_ = 4.88; *P* < 0.001), with the control treatment exhibiting significantly higher soil C:N ratios relative to the CO (2017: *P* < 0.01) and CB treatment (*P* < 0.01) (Table 1). However, there was no difference total C (2017: F_2,23_ = 0.47; *P* =0.63) nor total N (2017: F_2,23_ = 0.23; *P* = 0.8) among treatments.

*Microbial Biomass and Community Composition*

Analysis of variance also indicated a significant increase in microbial biomass N (F_2,23_ = 28.28; *P* = 0.001), within both the CO and CB treatments relative to the control (*P* < 0.001). In contrast, microbial biomass carbon did not differ (F_2,23_ = 1.76; *P* = 0.214). Given this, the MBC:N ratio significantly differed among treatments (F_2,23_ = 52.97; *P* < 0.001) with significant reductions in both the CO and CB treatments relative to the control (*P* < 0.001) (Supplementary Table 2).

Our preliminary data indicated that there was a significant effect of treatment on soil community composition (F_2, 37_ = 3.22; R^2^ = 0.15; *P* < 0.01), with CO and CB treatments exhibiting significantly different microbial communities as compared to control plots (*P* < 0.05) (Supplementary Fig. 3). Although there appeared to be separation between CO and CB treatment microbial community along the second axis, pairwise comparisons indicated non-significance (*P* = 0.058). Microbial PLFA abundances were significantly correlated with soil labile carbon (R^2^ = 0.25, *P* < 0.05), pH (R^2^ = 0.17, *P* < 0.05), soil C:N ratio (R^2^ = 0.228, *P* < 0.05), and DON (R^2^ = 0.215, *P* < 0.05) (Supplementary Table 4). Additionally, soil abiotic characteristics were significantly correlated with PLFA microbial community as indicated by the Mantel test (r = 0.231, *P* = 0.0009).

There was no difference in the relative biomass of primary microbial groups across treatments (Supplementary Table 3), except for gram-positive bacteria (F_2,23_ = 7.49; *P* < 0.01). Tukey HSD test indicated that the CB treatment had significantly more gram-positive bacteria than both the C (*p* < 0.001) and CO treatments (*P* < 0.01), while the CO treatment had elevated gram-positive abundances relative to the control but was non-significant (*P* = 0.06). In addition, there was also no difference in the ratio of fungi to bacteria (F_2,23_ = 0.389; *P* = 0.685).

SUPPLEMENTARY LIST OF FIGURES AND TABLES: PRELIMINARY STUDY

**Supplementary Figure S2:** Preliminary study principal coordinates axes of soil abiotic characteristics (pH, moisture, labile C, DOC, DON, DOC:N Ratio, inorganic N, total C:N Ratio) using Euclidean dissimilarities between samples. There was strong separation in soil abiotic characteristics in carrion addition plots relative to the control in the preliminary study conducted in the summer of 2017. Squares indicate controls (C), circles indicate carcass only (CO), and triangles indicate burying beetle plots (CB). Soil abiotic characteristics with a covariance greater than 0.3 were retained (indicated by arrows).

**
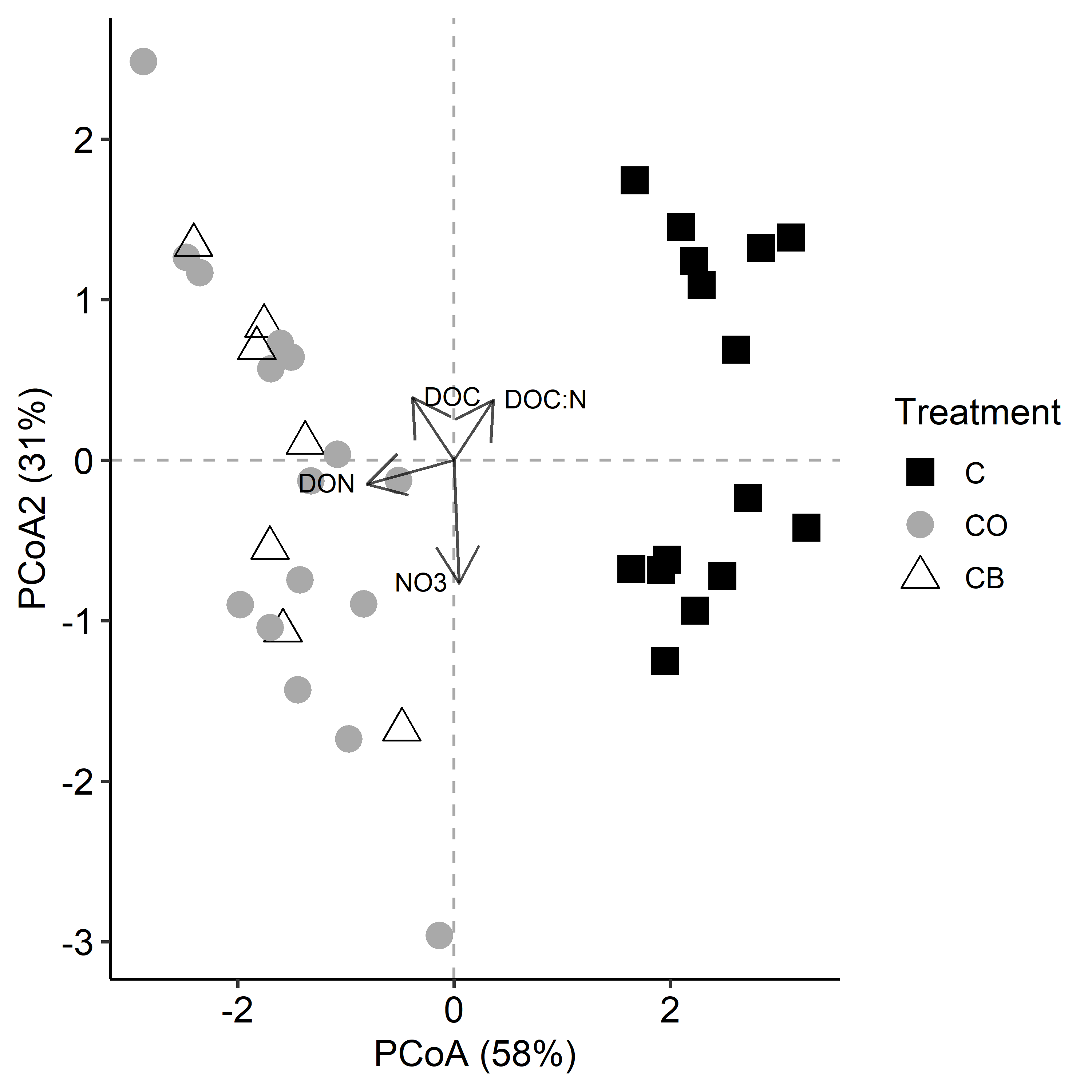
**

**Supplementary Figure S3:** Preliminary study NMDS ordination of microbial community abundances by treatment as measured by phospholipid fatty acid (PLFA) analysis in the summer of 2017. Carrion addition treatments differed relative to the control. Microbial PLFA abundances were significantly correlated with soil labile carbon and C:N ratio. Squares indicate control (C), circles indicate carcass only (CO), and triangles indicate burying beetle plots (CB). Soil abiotic characteristics with a correlation of 0.1 or greater were retained (indicated by arrows).


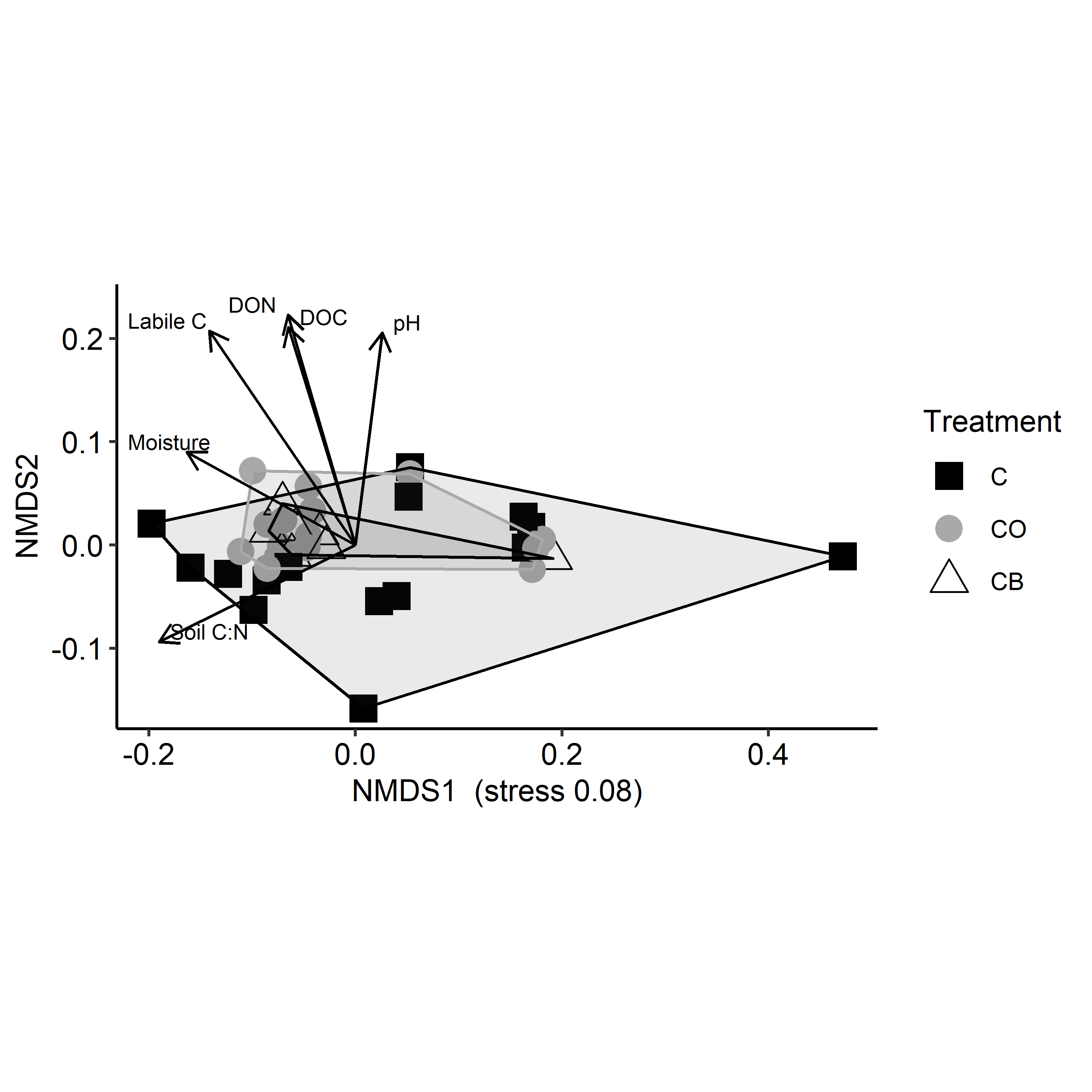


**Supplementary Table S2:** Preliminary study soil characteristics (means ± 1SE; n (2017): C = 15; CO = 15; CB = 7. Effects of treatment were tested with a one-way ANOVA. Treatment level differences were determined by Tukey-pairwise comparisons.

| **Treatment** | **Control (C)** | **Carcass Only (CO)** | **Carcass Burying Beetle (CB)** | **P-value** |
| --- | --- | --- | --- | --- |
| pH | 4.6 ± 0.16 | 5.9 ± 0.14 *** | 6.3 ± 0.16 *** | **< 0.001** |
| Moisture | 0.39 ± 0.04 | 0.56 ± 0.09 | 0.59 ± 0.08 | 0.253 |
| Labile C (µg C g^-1^ soil) | 2693 ± 299 | 4902 ± 938 | 5008 ± 753 | 0.088 |
| DOC (µg g^-1^ soil) | 811 ± 135 | 3992 ± 1486 *** | 3587 ± 983 *** | **< 0.01** |
| DON (µg g^-1^ soil) | 103 ± 13 | 3218 ± 244 *** | 3177 ± 342 *** | **< 0.001** |
| DOC:DON | 10.5 ± 1.46 | 1.19 ± 0.35 *** | 1.19 ± 0.23 *** | **< 0.001** |
| NH_4_^+^ (µg N g^-1^ soil) | 59 ± 3.99 | 40 ± 6.51 | 32 ± 6.64 * | **0.043** |
| NO_3_^-^ (µg N g^-1^ soil) | 2.8 ± 0.81 | 3.6 ± 1.29 | 3.3 ± 1.69 | 0.781 |
| Total C (%) | 17.9 ± 2.9 | 15.7 ± 2.52 | 12.8 ± 1.56 | 0.634 |
| Total N (%) | 0.84 ± 0.11 | 0.93 ± 0.12 | 0.81 ± 0.085 | 0.801 |
| C:N | 19.9 ± 0.94 | 16.2 ± 0.61 ** | 15.9 ± 0.78 ** | **< 0.001** |
| MBC (µg g^-1^ soil) | 2330 ± 278 | 3145 ± 384 | 3984 ± 750 | 0.214 |
| MBN (µg g^-1^ soil) | 394 ± 49 | 2553 ± 591 *** | 2659 ± 361 *** | **< 0.001** |
| MBC:N | 7.0 ± 0.37 | 1.7 ± 0.14 *** | 1.9 ± 0.32 *** | **< 0.001** |

* significant at *P* < 0.05; ** *P* < 0.01; *** *P* < 0.001 relative to the control.

**Supplementary Table S3:** Preliminary study **s**oil microbial biomass as estimated by phospholipid fatty acid (PLFA) analysis (nmol g*^-1^* dry soil) (means ± 1SE; n (2017): C = 15, CO = 15, CB = 7; Effects of treatment were tested with a one-way ANOVA.

| **Treatment** | **Control (C)** | **Carcass Only (CO)** | **Carcass Burying Beetle (CB)** | **P-value** |
| --- | --- | --- | --- | --- |
| *2017* |  |  |  |  |
| Total microbial biomass | 236 ± 26 | 260 ± 15 | 378 ± 51 | 0.063 |
| Fungi | 15.6 ± 1.3 | 16.8 ± 1.6 | 24.7 ± 3.8 | 0.091 |
| Bacteria | 204 ± 25 | 225 ± 13 | 326 ± 43 | 0.068 |
| Gram-negative bacteria | 138 ± 22 | 141 ± 9 | 193 ± 60 | 0.256 |
| Gram-positive bacteria | 65.1 ± 4.7 | 83.4 ± 5.4 | 131.9 ± 19.3 *** ^●●^ | **0.007** |
| Fungi:bacteria Ratio | 0.081 ± 0.001 | 0.073 ± 0.004 | 0.074 ± 0.004 | 0.686 |

* significant at *P* < 0.05; ** *P* < 0.01; *** *P* < 0.001 relative to the control.

^●^ significant at *P* < 0.05; ^●●^  *P* < 0.01; ^●●●^ *P* < 0.001 relative to carcass only treatment.

**Supplementary Table S4:** Preliminary study soil abiotic attribute correlations with NMDS ordination axes.

|  | **2017 Environmental Correlations** | | | |
| --- | --- | --- | --- | --- |
| **Response Variable** | *NMDS1* | *NMDS2* | *r2* | *pr(>r)* |
| Labile C | -0.563 | 0.826 | 0.252 | **0.035 *** |
| NO_3_^-^ | 0.259 | -0.965 | 0.067 | 0.303 |
| NH_4_^+^ | -0.021 | -0.99 | 0.080 | 0.265 |
| Moisture | -0.875 | 0.482 | 0.139 | 0.091 |
| pH | 0.124 | 0.992 | 0.171 | **0.033 *** |
| Soil C:N | -0.897 | -0.442 | 0.179 | **0.030 *** |
| DOC | -0.295 | 0.955 | 0.195 | 0.064 |
| DON | -0.281 | 0.959 | 0.215 | **0.015 *** |
| DOC:DON | -0.018 | -0.999 | 0.050 | 0.395 |
